# Incorporating Antimicrobial Resistance Considerations in Antimicrobial Evidence‐Based Practice Guidelines: A Protocol for the Development of a Checklist and Multifaceted Appraisal of Existing Guidelines

**DOI:** 10.1002/gin2.70057

**Published:** 2026-01-29

**Authors:** Sara Ibrahim, Mark Loeb, Jessica Bartoszko, Maryam Ghadimi, Gordon Guyatt, Benedikt Huttner, Alfonso Iorio, Dominik Mertz, Lorenzo Moja, Gabriel Rada, Pilar Ramon‐Pardo, Ludovic Reveiz, Gerard Wright, Nancy Santesso, Romina Brignardello‐Petersen

**Affiliations:** ^1^ Department of Health Research Methods, Evidence, and Impact McMaster University Hamilton Canada; ^2^ Department of Pathology & Molecular Medicine McMaster University Hamilton Canada; ^3^ Department of Medicine McMaster University Hamilton Canada; ^4^ Antimicrobial Resistance Division World Health Organization Geneva Switzerland; ^5^ Health Products Policy and Standards World Health Organization Geneva Switzerland; ^6^ Epistemonikos Foundation Santiago Chile; ^7^ Universidad del Desarrollo Santiago Chile; ^8^ Pan American Health Organization Washington DC USA; ^9^ Department of Biochemistry & Biomedical Sciences, David Braley Center for Antibiotic Discovery, Michael G. DeGroote Institute for Infectious Disease Research McMaster University Hamilton Canada

**Keywords:** antimicrobial resistance, critical appraisal, guideline development

## Abstract

**Introduction:**

Antimicrobial resistance (AMR) is a widely recognized threat to global health, mainly driven by the inappropriate use of antimicrobials. Recommendations from evidence‐based practice guidelines (EBPGs) can promote appropriate antimicrobial use. Currently, no guidance exists for considering AMR in EBPGs. This is the protocol for two studies that aim to (1) develop a checklist of AMR considerations for EBPGs and (2) conduct a case study to evaluate existing EBPGs regarding the extent to which AMR is considered among other methodological assessments.

**Methods:**

Using the Delphi technique, we will consult with individuals from four interest‐holder groups to develop and refine a checklist of AMR considerations for EBPGs. To assess EBPGs, we will search various databases for guidelines on four prioritized syndromes (i.e., community‐acquired pneumonia, urinary tract infection, skin and soft tissue infections and exacerbations of chronic obstructive pulmonary disease). We will screen articles in duplicate and in stages to include EBPGs for the prioritized syndromes and will use piloted data extraction forms to extract characteristics of the EBPGs. For each EBPG, we will evaluate whether AMR was considered (using the developed checklist), its trustworthiness (i.e., methodological quality) (using the modified National Guideline Clearinghouse Extent of Adherence to Trustworthy Standards instrument) and its adaptability (using items from existing adaptability frameworks).

**Discussion:**

This work will develop a checklist of AMR considerations for EBPGs and evaluate a subsequent sample of EBPGs to investigate the extent to which AMR is considered. Furthermore, by conducting a methodological appraisal of trustworthiness and adaptability, this study will contextualize results to promote the uptake and appropriate interpretation of existing guidelines. Thus, these findings will be useful to decision‐makers as well as guideline development groups as they highlight what existing work can be trusted and leveraged while also identifying what limitations and gaps need to be addressed.

## Summary

- Recommendations from evidence‐based practice guidelines that consider antimicrobial resistance (AMR) can promote appropriate antimicrobial use, which is key to addressing AMR.
- Currently, no guidance exists for explicitly considering AMR during guideline development, and it is unclear how and to what extent guideline development groups are doing this in practice.
- This protocol outlines a two‐stage project that involves: (1) the development of a checklist of AMR considerations for the planning, development and reporting of guidelines, and (2) a scoping review and critical appraisal of existing guidelines of four syndromes to evaluate their methodological quality, adaptability and extent to which AMR is considered.

## 1 Introduction

Antimicrobial resistance (AMR) is a well‐recognized threat to global public health driven by various factors [1, 2]. Aside from its serious health consequences, AMR is associated with large costs to the economy and health system [1, 3, 4]. Although a natural process over time, the emergence and spread of AMR is exacerbated by human activity, such as misuse and overuse of antimicrobials [1, 2]. One strategy to address this is antimicrobial stewardship: a system‐wide approach that includes interventions aimed at promoting, improving, monitoring and evaluating appropriate antimicrobial use [5, 6]. One example of a stewardship tool that can be implemented at the global, national or regional level is evidence‐based practice guidelines (EBPGs).

EBPGs provide actionable recommendations regarding the most appropriate intervention and are thus—when they consider AMR—aimed at optimising and promoting appropriate antimicrobial use. It is unclear, however, how and to what extent guideline development groups (GDGs) consider AMR when developing their recommendations. Thus, this protocol outlines the methods we will use for determining whether and how EBPGs address relevant AMR considerations, and to promote the appropriate interpretation and uptake of these guidelines. We describe two studies, one focused on the development of a checklist of AMR considerations for the development and assessment of EBPGs, and another describing the methods for selecting and critically appraising a sample of AMR‐related EBPGs to guide uptake of existing recommendations and future guideline development efforts.

## 2 Methods

**Figure 1:**
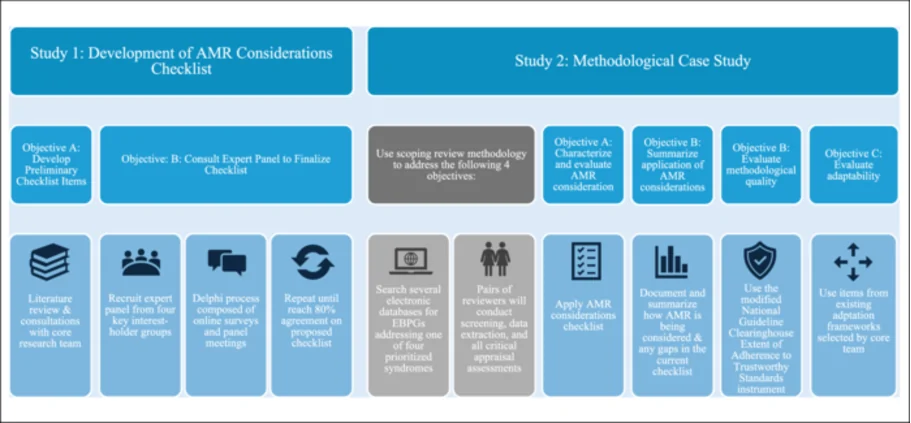
Overview of the two studies outlined in this protocol. AMR, antimicrobial resistance; EBPG, evidence‐based practice guideline.

We will develop a checklist of AMR considerations for EBPGs (Study 1) and conduct a methodological study to assess a sample of existing EBPGs (Study 2). Figure 1 provides an overview of both studies. Any protocol changes will be communicated in the final publication of each study.

We used the DELPHISTAR to guide the reporting of our protocol for the first study and adhered to the reporting guidance for scoping review protocols for the second [7, 8]. The Supporting Appendix includes a list of terms and definitions relevant to our methods.

### 2.1 Study 1: Development of a Checklist of AMR Considerations for EBPGs

Objectives:

A. To develop a list of preliminary checklist items related to AMR considerations that EBPGs of antimicrobial therapies should address.
B. To ensure comprehensiveness and clarity of the checklist content by consulting various interest‐holders.

#### 2.1.1 Development of Preliminary Checklist Items (Objective A)

The checklist will aim to address AMR‐specific considerations for GDGs developing EBPGs with AMR implications (e.g., treatment of bacterial infections in humans). Although the scope of the considerations includes the planning and development, in addition to the reporting of the EBPG, our methods for developing the checklist will be based on the guidance for developing reporting guidelines [9]. Through a literature review, we will identify existing development frameworks, reporting guidelines, examples of EBPGs that have explicitly considered AMR, or any other documents addressing AMR considerations in EBPGs. We will discuss these findings with our interdisciplinary core research team, which includes EBPG methodologists, AMR experts and experts who develop AMR‐related EBPGs, to assist with generating potential checklist items. To organize preliminary items, we will map each of the considerations relevant to EBPGs development to domains and items of the GIN‐McMaster guideline development checklist [10] and the considerations relevant to reporting to the AGREE Reporting checklist [11]. We will identify which considerations are relevant to the whole guideline versus specific recommendations.

#### 2.1.2 Revision of Items Based on Interest‐Holder Consultation (Objective B)

Interest‐holders will revise the preliminary items and assess their comprehensiveness and relevance. To consider the perspective of all relevant groups, we will recruit an expert panel from across the globe composed of: (1) EBPG methodologists, (2) AMR experts, (3) end‐users of AMR‐related EBPGs (e.g., clinicians, researchers, public health organizations, policy‐makers) and (4) end‐users of our checklist (e.g., members of EBPGs oversight committees, members of GDGs). We aim to recruit a total of 20 participants (5 experts/group) and will rely on the networks of our research team and collaborators to invite participants and disseminate recruitment information. We will use purposive sampling to have a diverse group of experts that includes representation of various genders and geographic regions.

We will use the Delphi technique to gather the opinions of our expert panel and to reach consensus regarding the most relevant items to include in our checklist [12, 13]. First, using LimeSurvey, panellists will rate the importance of each of the preliminary checklist items using a numeric rating scale from 1 (*not important*) to 7 (*extremely important*) [14]. To ensure having a comprehensive checklist, panellists may suggest any additional items they believe should be included and to suggest any clarifications to the initial items. Thus, the first round will focus on clarifying and identifying relevant checklist items and the elimination of any items with a median score of 2 or less and for which no panellists rated as at least a 6. Subsequently, the next round of the survey will incorporate these revisions. Survey data will be aggregated and anonymized, and the research team will only be aware of each participant's response status (complete or incomplete) in case follow‐ups are needed.

We will present the survey results (i.e., descriptive statistics of the overall rating per item) and a suggested checklist of items developed by the research team based on the panel ratings at a virtual panel meeting. We will obtain consent from panellists to record this meeting and take detailed meeting minutes. Here, panellists will discuss the suggested checklist, the core team will incorporate decisions, and, using an anonymous poll, the panel will vote as to whether they agree with the full list. If a panel member cannot attend the meeting, they will provide their vote via email. If we do not reach consensus (defined as 80% agreement) at the first meeting, panel members will respond to another round of survey focusing on the items that are the source of the disagreement, per the meeting discussion, and will discuss the new suggested checklist based on the latest survey results in another meeting. We will repeat this process until consensus is reached and a final checklist (with a maximum of 15 items to ensure feasibility in subsequent objectives) is developed. Our report will include all potential items generated in our findings and describe which items were retained and which were not, with reasons for the excluded items. We will assess the face validity of our finalized checklist by presenting it to relevant experts at conferences (e.g., through workshops). The DELPHISTAR checklist [8] will guide the reporting of this process.

### 2.2 Study 2: Critical Appraisal of a Sample of Existing EBPGs With AMR Implications

Objectives:

A. To classify, characterise and evaluate EBPGs according to our identified AMR considerations.
B. To summarize how AMR is currently being considered in EBPGs and conduct pilot testing of the checklist developed in Study 1.
C. To evaluate the trustworthiness (i.e., methodological quality) of these EBPGs.
D. To evaluate the adaptability of these EBPGs.

To address these, we will conduct a methodological review using the scoping review framework outlined by the Joanna Briggs Institute [15].

#### 2.2.1 Search Strategy

An information specialist at the McMaster Health Information Research Unit will: (1) develop a comprehensive search strategy for both general databases (i.e., Medline and Embase accessed through the Ovid platform) and guideline‐specific databases (i.e., Guidelines International Network library, Turning Research Into Practice), and (2) search other literature sources (e.g., Public Health Agency of Canada, United States Centre for Disease Control and Prevention), including the 25 international websites searched in a previous systematic survey of infectious diseases guidelines [16]. We will consult with content experts for any additional publications. Searches will not be restricted by date but, for feasibility, will only include EBPGs published in English.

#### 2.2.2 Eligibility Criteria

Because any EBPGs pertaining to antimicrobial usage can have AMR implications, we will include any EBPGs (defined as a guideline informed by a systematic review, see Supporting Information for complete definition) addressing at least one of four prioritized syndromes: community‐acquired pneumonia, urinary tract infections, skin and soft tissue infections and exacerbations of chronic obstructive pulmonary disease. These are among the most common and serious syndromes in the community and were previously prioritized by the World Health Organization's essential medicines group [17]. To determine whether recommendations were informed by a systematic review, we will rely on either: (1) the author's definition or (2) the use of established criteria to determine the eligibility of studies. Because AMR is accelerated by the overuse and misuse of antimicrobials, to be eligible, EBPGs will need to include at least one recommendation (see Supporting Information for definition) relevant to such interventions [1, 2].

#### 2.2.3 Study Selection

Using Covidence, we will screen publications in duplicate and in stages: titles/abstracts and full‐texts [18]. Before screening, all reviewers will receive training and complete a calibration exercise. Discrepancies will be resolved through discussion or by a third reviewer.

#### 2.2.4 Data Extraction

Following training and calibration, we will extract data from all eligible EBPGs using piloted, standardized extraction forms, independently and in duplicate. We will resolve discrepancies through discussion or by consulting a third reviewer. The Supporting Information summarizes all data extraction variables across objectives.

For *Objective A*, data extraction will include a variety of information identified as relevant for methodological studies [19], and information related to the general characteristics of the guideline, the guideline topic and the recommendations themselves. Next, to assess the extent to which each EBPG considered AMR, we will apply the checklist developed in Study 1 to determine whether AMR was considered at both the guideline level and for each recommendation, as it may vary.

For *Objective B*, data extraction will include details related to how AMR was considered and reported alongside any other examples of AMR being considered not captured by the checklist. Additionally, reviewers will provide feedback regarding any difficulties applying the checklist that may be derived from the wording of items. Such data will be useful for improving our preliminary checklist, including clarifying items to address any ambiguity, and informing future efforts regarding the methods for applying our list of considerations.

For *Objective C*, the modified version of the National Guideline Clearinghouse Extent of Adherence to Trustworthy Standards (NEATS) instrument will be used to assess trustworthiness [20]. The modified NEATS aims to improve the reproducibility of assessments and ensure items evaluate the appropriateness of the guideline development process rather than reporting quality [20]. The tool includes ten items assessing transparent disclosure of funding, management of conflicts of interest, composition of the GDG, systematic review process, rating the strength of recommendations, articulation of recommendations, composition of the external review team and plans for updating [20]. Extraction fields will follow all steps of the assessment algorithms outlined in the instrument manual to reach a final rating from 1 (*no information to low methodological quality*) to 5 (*high methodological quality*) for eight of the items and a rating of yes/no for the remaining two items [20].

For *Objective D*, items from existing adaptation frameworks such as the GRADE‐ADOLOPMENT, MAGIC and ADAPTE frameworks will guide our adaptability assessments [21]. We will identify items from existing frameworks that are specific to identifying appropriate source guidelines, including relevancy (e.g., consider topic, country and language of publication), trustworthiness (based on assessment in Objective C), currency (e.g., last date of search for relevant systematic reviews), availability of relevant evidence syntheses and availability of justification of judgments in GRADE evidence‐to‐decision tables or others [22, 23].

Making the assumption that guidelines that include multiple recommendation statements use the same methods to develop all recommendations, we will conduct the assessments for Objectives C and D at the guideline level. For any item that needs to be assessed at the recommendation level, we will assess only the first recommendation and apply this rating to the entire guideline.

#### 2.2.5 Data Analysis and Presentation

The Scoping Reviews extension of the Preferred Reporting Items for Systematic Reviews and Meta‐analyses checklist [24] will guide the reporting of our findings.

For *Objective A*, we will: (1) summarize the features of the included EBPGs in tables, including details regarding the year, country, topic characteristics (e.g. population, scope) and ways in which AMR was considered, (2) use descriptive statistics to calculate the frequency of EBPGs published per syndrome and the percentage of EBPGs and recommendations meeting each of our checklist criteria, (3) calculate the median number of checklist items met per EBPG and per recommendation, (4) categorize EBPGs according to overlap within their questions and summarize their recommendations, (5) assess whether recommendations varied across guidelines according to whether AMR was considered or according to different characteristics that can impact health opportunities and outcomes (e.g., sex, country economy) and (6) calculate the frequency with which recommendations considered the PROGRESS‐Plus characteristics [25].

For *Objective B*, using an inductive content analysis approach, we will categorize the methods by which current EBPGs consider AMR [26]. This will involve reviewing the extracted data to conduct open coding, the results of which will be used to develop a coding framework (through discussions with the research team) [26]. We will then apply this framework to the data, which can be used to inform future efforts regarding how to apply our list of AMR considerations [26].

For *Objective C*, we will use descriptive statistics and tables to summarize the score across the sample of EBPGs for each of the ten NEATS items. Additionally, we will calculate the proportion of EBPGs with acceptable adherence (defined as ‘Yes’ on categorical items or at least a 4 on items scored on a Likert scale) for each of the ten NEATS items [20].

For *Objective D*, we will summarize the number of adaptability items met per EBPG using descriptive statistics and tables. We will map the EBPGs according to both their trustworthiness and adaptability (from most to least) and identify any common limitations across EBPGs.

### 2.3 Ethics and Dissemination

We will seek ethics approval from the Hamilton Integrated Research Ethics Board for Study 1 and will require all participants to provide informed consent. Ethics approval and consent are not required for Study 2 as we will use published documents.

We will disseminate our findings through traditional knowledge translation routes by publishing our results while adhering to existing reporting guidelines [8, 27]. The final outputs of this work will include a checklist of AMR considerations for guideline development (Study 1) and a methodological scoping review of existing antimicrobial guidelines characterizing them with respect to their trustworthiness, adaptability and the extent to which they considered AMR (Study 2). Additionally, we will disseminate our findings through the development of an open‐access, user‐friendly online database, for which a protocol, describing our methods for user‐testing, will be published at a later stage.

## 3 Discussion

As AMR evolves, identifying the extent to which AMR is appropriately considered in EBPGs, how trustworthy these EBPGs are, and any room for improvement and adaptation of such EBPGs is important for supporting the use of current EBPGs and the development of future ones. This work aims to achieve these aims by: (1) consulting with both AMR and guideline methodology experts to develop a checklist of AMR considerations for guideline development, and (2) conducting a scoping review to evaluate existing EBPGs within four prioritized syndromes to determine the extent to which AMR is being considered in practice, alongside assessments of trustworthiness and adaptability.

To our knowledge, this work is the first of its kind, as currently no guidance exists for considering AMR in the planning, development and reporting of guidelines. This work builds upon a previous systematic survey of AMR considerations in primarily the recommendations of tuberculosis, gonorrhoea and respiratory tract infection guidelines published before mid‐2019 by: (1) formally developing a checklist of AMR considerations for the entire guideline development process which can be used to assess existing guidelines and assist with developing future guidelines, and (2) applying this checklist to a sample of EBPGs of different syndromes to evaluate and summarize the extent to which AMR is currently being considered [16]. Furthermore, by contextualizing our findings with respect to trustworthiness and adaptability, this work can facilitate the uptake of guidelines while equipping GDGs with information regarding what gaps may exist and what existing work can be leveraged in the development of further guidance to promote appropriate antimicrobial use to address AMR, saving time and other resources. Although this work will provide insights on which AMR considerations are important and describe how AMR is currently being considered in EBPGs, it will not develop formal frameworks for how to appropriately apply these considerations, nor will it provide explicit guidance on how to best promote the implementation of guidelines.

### 3.1 Strengths and Limitations

The strengths of our study include adherence to rigorous, systematic methodological standards, such as conducting a comprehensive search in several databases, using prespecified eligibility criteria and minimizing errors by conducting all stages in duplicate. Furthermore, the use of piloted extraction, calibration exercises and the NEATS manual assists with improving the consistency and reproducibility of our findings. Due to feasibility, limitations of this work include restricting the sample to EBPGs published in English, which may contribute to language bias [28, 29]. Additionally, another limitation is our case study design, which is limited to four syndromes based on resources. However, by transparently reporting our methods and results, this work can be expanded to larger samples as needed. Lastly, despite following guidance for developing reporting tools to inform the development of our checklist, item generation is limited to a targeted literature search and discussions with experts, and the validation component is limited to face validity [9]. Thus, the results of our preliminary checklist, which to our knowledge would be the first of its kind, can serve as a basis for the development and validation of formal methodological and reporting guidelines for AMR considerations in EBPGs, and the findings from our second study can also help further improve this checklist in the future.

## Author Contributions

**Sara Ibrahim:** conceptualization, writing – original draft, writing – review and editing, methodology, funding acquisition, project administration. **Mark Loeb:** conceptualization, writing – review and editing, funding acquisition, supervision, methodology. **Jessica Bartoszko:** conceptualization, writing – review and editing, methodology. **Maryam Ghadimi:** writing – review and editing, methodology. **Gordon Guyatt:** writing – review and editing, methodology. **Benedikt Huttner:** writing – review and editing, methodology. **Alfonso Iorio:** writing – review and editing, methodology. **Dominik Mertz:** writing – review and editing, methodology. **Lorenzo Moja:** writing – review and editing, methodology. **Gabriel Rada:** writing – review and editing, methodology. **Pilar Ramon‐Pardo:** writing – review and editing, methodology. **Ludovic Reveiz:** writing – review and editing, methodology. **Gerard Wright:** writing – review and editing, methodology. **Nancy Santesso:** conceptualization, writing – review and editing, methodology. **Romina Brignardello‐Petersen:** conceptualization, writing – review and editing, supervision, methodology, funding acquisition.

## Disclosure

Stage of the project: at the time of submission, the authors had not begun any stage of the project. At the time of peer‐review, they had completed recruitment of the expert panel, both rounds of the survey, and hosted two panel meeting to discuss the checklist and were in the process of finalizing the list of items for inclusion for Study 1. They have not begun any stage of Study 2.

## Conflicts of Interest

All authors have completed the ICMJE uniform disclosure form at www.icmje.org/coi_disclosure.pdf and declare: support from the Canadian Institutes of Health Research; M.L. is part of an advisory board for Xediton Pharmaceuticals and is involved in AMMI‐Canada Working Group on Antibiotic Guidelines and WHO EML Antibiotic Working Group; J.B. has received contracts from the WHO and is employed by the Public Health Agency of Canada—however this work is unrelated to J.B.'s employment responsibilities; G.W. is a member of the Public Health Agency of Canada Expert Advisory Board for Antimicrobial Resistance and reports stock ownership in Prokaryotics Inc, Kapoose Creek Bio and Claryx Inc. The remaining authors declare no financial relationships with any organisations that might have an interest in the submitted work in the previous 3 years; no other relationships or activities that could appear to have influenced the submitted work. The authors alone are responsible for the findings, conclusions, and views expressed in this publication, and they do not necessarily represent the decisions or policies of the Pan American Health Organization, the official position of the WHO, or any of the institutions mentioned.

## Supporting information

Supplementary Material.

## Data Availability

The authors have nothing to report.
