# Supplementary material for: Incorporating Antimicrobial Resistance Considerations in Antimicrobial Evidence‐Based Practice Guidelines: A Protocol for the Development of a Checklist and Multifaceted Appraisal of Existing Guidelines
Source: Clin Public Health Guidel. 2026 Jan 29;3(2):e70057. doi: 10.1002/gin2.70057 (PMC13563331; doi:10.1002/gin2.70057)
Supplement: Supplementary file 1 [file GIN2-3-e70057-s001.docx]

Supplementary Material

Table of Contents

Table 1: Terms and Definitions ..................................................................................... 1

Table 2: Proposed Data Extraction Fields ..................................................................... 2

| Table 1: Terms and Definitions | |
| --- | --- |
| Evidence-based practice guideline (EBPG) | The entirety of a document containing a series of healthcare recommendations for optimizing patient care that are informed by a systematic review of the existing evidence.^1^ |
| Recommendation | A single actionable statement that communicates which of two or more intervention options should be used for a specified group of patients.^2^ |
| AMR considerations | Factors that anticipate or account for potential AMR influence or consequences that guideline development groups should consider when developing and publishing EBPGs and recommendations related to the use of antimicrobials. |
| Trustworthiness (i.e., methodological quality) | The extent to which users can be confident that an EBPG was developed using rigorous methods and whose recommendations can be relied on.^3^ |
| Adaptability | The degree to which an existing EBPG can be modified to apply to a different context (instead of generating a new EBPG).^4^ |

1. Institute of Medicine (US) Committee on Standards for Developing Trustworthy Clinical Practice Guidelines. *Clinical Practice Guidelines We Can Trust*. (Graham R, Mancher M, Miller Wolman D, Greenfield S, Steinberg E, eds.). National Academies Press (US); 2011. Accessed January 30, 2023. <http://www.ncbi.nlm.nih.gov/books/NBK209539/>
2. Lotfi T, Hajizadeh A, Moja L, et al. A taxonomy and framework for identifying and developing actionable statements in guidelines suggests avoiding informal recommendations. *J Clin Epidemiol*. 2022;141:161-171. doi:10.1016/j.jclinepi.2021.09.028
3. Ghadimi M, Guyatt G, Brignardello-Petersen R. Modifications to the NEATS instrument for more appropriate and reproducible assessment of guidelines trustworthiness. *Clin Public Health Guidel*. 2024;1(3):e12025. doi:10.1002/gin2.12025
4. Darzi A, Abou-Jaoude EA, Agarwal A, et al. A methodological survey identified eight proposed frameworks for the adaptation of health related guidelines. *J Clin Epidemiol*. 2017;86(Complete):3-10. doi:10.1016/j.jclinepi.2017.01.016

| Table 2: Proposed Data Extraction Fields | |
| --- | --- |
| General Characteristics | - Title of the EBPGs - Last name of the first author - Year of publication - Guideline organization - Country of guideline organization or country of authors - Source of Funding - Language(s) of publication - Type of guideline (de novo, update, adapted, living) - Context of guidelines (national, international) - Prioritized syndrome (community-acquired pneumonia, urinary tract infections, skin and soft tissue infections, and exacerbations of chronic obstructive pulmonary disease) |
| Guideline Methods | - Target audience and scope - Number of health care questions addressed - Extract details related to the specific population, interventions, and outcomes addressed   - Including PROGRESS-Plus^26^ characteristics (e.g., sex, socioeconomic status, social capital, and country economy) - Guideline development approach (e.g., GRADE, SIGN, etc) - Guideline panel size and composition - Inclusion of patient partners in guideline development (yes/no) - Systematic review search dates - Reporting of systematic review findings - Location (e.g., appendix, associated publication, unpublished) - Presence of reporting of key results (yes/incomplete/no)   - Risk of bias assessment   - Meta-analyses   - Summary of findings tables |
| Guideline Results | - Number of recommendations formulated - Verbatim extraction of each recommendation statement - Extract the quality of the evidence per recommendation - Extract the strength of each recommendation - Consideration of PROGRESS-Plus^26^ characteristics in when developing recommendations (e.g. sex, socioeconomic status, social capital, and country economy) - Reporting of evidence-to-decision judgements per recommendation (yes/incomplete/no)   - Location (e.g., appendix, associated website) |
| Critical appraisal | - AMR considerations at the guideline level and for each recommendation   - Extract per checklist item at both the guideline and recommendation level, whether the consideration was met or not and provide a justification   - When AMR considerations are met, extract specific example including verbatim quotes at both the guideline and recommendation level     - Extract location of reporting within guideline   - Extract verbatim quotes of any additional examples of AMR being considered not currently captured by the checklist - Assessment of trustworthiness according to NEATS   - Extract per NEATS signalling question the judgement and subsequent final rating per item with a justification - Assessment of adaptability   - Extract per adaptability item the degree of adaptability (yes, probably yes, probably no, no) and provide a justification |
